# Knowledge Mapping of Exosomes in Autoimmune Diseases: A Bibliometric Analysis (2002–2021)

**DOI:** 10.3389/fimmu.2022.939433

**Published:** 2022-07-22

**Authors:** Fengping Wu, Jinfang Gao, Jie Kang, Xuexue Wang, Qing Niu, Jiaxi Liu, Liyun Zhang

**Affiliations:** ^1^ School of Basic Medical Sciences, Shanxi Medical University, Taiyuan, China; ^2^ Department of Rheumatology, Shanxi Bethune Hospital, Shanxi Academy of Medical Sciences, Tongji Shanxi Hospital, Third Hospital of Shanxi Medical University, Taiyuan, China; ^3^ Third Hospital of Shanxi Medical University, Shanxi Bethune Hospital, Shanxi Academy of Medical Sciences, Tongji Shanxi Hospital, Taiyuan, China

**Keywords:** bibliometrics, exosomes, autoimmune diseases, CiteSpace, VOSviewers

## Abstract

**Background:**

Autoimmune diseases (AIDs) are a class of chronic disabling diseases characterized by inflammation and damage to muscles, joints, bones, and internal organs. Recent studies have shown that much progress has been made in the research of exosomes in AIDs. However, there is no bibliometric analysis in this research field. This study aims to provide a comprehensive overview of the knowledge structure and research hotspots of exosomes in AIDs through bibliometrics.

**Method:**

Publications related to exosomes in AIDs from 2002 to 2021 were searched on the web of science core collection (WoSCC) database. VOSviewers, CiteSpace and R package “bibliometrix” were used to conduct this bibliometric analysis.

**Results:**

312 articles from 48 countries led by China and the United States were included. The number of publications related to exosomes in AIDs is increasing year by year. Central South University, Sun Yat Sen University, Tianjin Medical University and University of Pennsylvania are the main research institutions. *Frontiers in immunology* is the most popular journal in this field, and *Journal of Immunology* is the most co-cited journal. These publications come from 473 authors among which Ilias Alevizos, Qianjin Lu, Wei Wei, Jim Xiang and Ming Zhao had published the most papers and Clotilde Théry was co-cited most often. Studying the mechanism of endogenous exosomes in the occurrence and development of AIDs and the therapeutic strategy of exogenous exosomes in AIDs are the main topics in this research field. “Mesenchymal stem cells”, “microRNA”, “biomarkers”, “immunomodulation”, and “therapy” are the primary keywords of emerging research hotspots.

**Conclusion:**

This is the first bibliometric study that comprehensively summarizes the research trends and developments of exosomes in AIDs. This information identifies recent research frontiers and hot directions, which will provide a reference for scholars studying exosomes.

## Introduction

AIDs are a class of diseases caused by the loss of the body’s autoimmune tolerance leading to damage to its own tissues, affecting about 10% of the world’s population (1). AIDs are characterized by inflammation and damage to muscles, joints, bones, and internal organs, including rheumatoid arthritis (RA), systemic lupus erythematosus (SLE), and type 1 diabetes (T1D), Sjögren’s Syndrome (SS), Multiple Sclerosis (MS) and more than 80 diseases. At present, the treatment of AIDs is mainly based on anti-rheumatic drugs such as non-steroidal anti-inflammatory drugs, hormones, and biological agents. However, long-term use of these drugs can cause many side effects, so it is imperative to find safe and effective alternatives (2).

Exosomes are extracellular vesicles (EVs) with a diameter of about 30~150 nm, and their contents are rich, including: DNA, RNA, lipids, proteins, cytokines and other substances. The other two extracellular vesicles with larger diameters are microvesicles and apoptotic bodies, but the immunomodulatory effect of exosomes is the strongest (3, 4). In addition, the source of exosomes is wide, and their parental cells are diverse, including: mesenchymal stem cells (MSCs), Dendritic cells (DC), T cells, etc. Compared with parent cells, exosomes have the characteristics of stable physicochemical properties, no heterologous risk, and easy storage and transportation, so they are likely to be ideal products for the treatment of AIDs in the future (5, 6). In recent years, many scholars have begun to try to use exosomes to treat AIDs and have made great breakthroughs (6–13), and related clinical trials have gradually been launched (14).

Bibliometrics is a literature analysis method that analyzes the output and status of publications in a particular research field from a quantitative and qualitative perspective (15, 16). During the analysis, we can obtain detailed information about authors, keywords, journals, countries, institutions, references, etc. in the relevant research field (16). Bibliometric tools such as CiteSpace (17), VoSviewer (18), R package” bibliometrix” (19), and HistCite (20) are commonly applied to visualize the results of literature analysis, and these tools have been widely used in medical fields, such as oncology (21, 22), Orthopedics (23), Thoracic Surgery (24), and Rheumatology (25). In 2019, Wang B et al. summarized the research status of exosomes in the world, but the study did not introduce the detailed research progress of exosomes in various medical disciplines (15). As far as we know, although related scholars have done bibliometric studies of exosomes in cancer (21), cardiovascular disease (26), and diabetes (27), studies of exosomes in AIDs have not emerged by means of bibliometrics. However, related studies in recent years have shown that exosomes show good prospects in the treatment of AIDs. To fill this knowledge gap, this study aimed to perform a bibliometric analysis of publications of exosomes in AIDs over the past two decades (from 2002 to 2021) to identify major contributors and current research status, and to look forward to the research trends and future development prospects in this field.

## Methods

### Search Strategy

**Figure 1:**
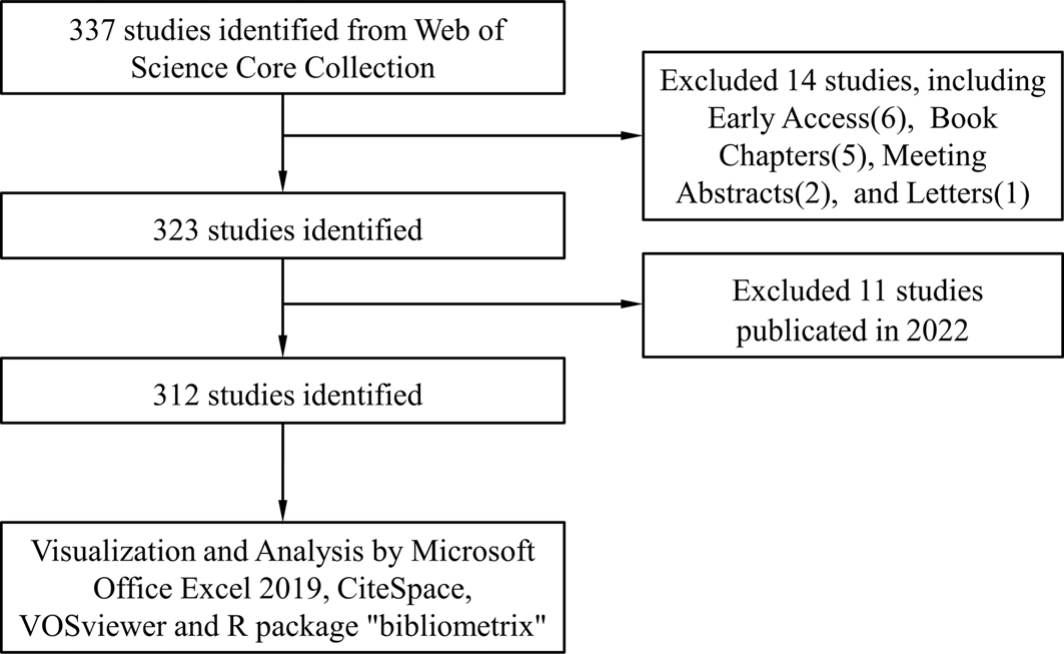
Publications screening flowchart.

We conducted a literature search on the Web of Science Core Collection (WoSCC) database (https://www.webofscience.com/wos/woscc/basic-search) on 15 April 2022. The search formula is ((TS = (Exosomes)) AND TS = (Autoimmune Diseases)) AND LA = (English), and the type of documents is set to “articles “ and “review” (**Figure 1**).

### Data Analysis

VOSviewer (version 1.6.18) is a bibliometric analysis software that can extract the key information from numerous publications (28), which is often used to build collaboration, co-citation and co-occurrence networks (29, 30). In our study, the software mainly completes the following analysis: country and institution analysis, journal and co-cited journal analysis, author and co-cited author analysis, and keyword co-occurrence analysis. In the map produced by VOSviewer, a node represents an item such as country, institution, journal, and author. Node size and color indicate the number and classification of these items, respectively. Line thickness between nodes reflects the degree of collaboration or co-citation of the items (25, 31).

CiteSpace (version 6.1.R1) is another software developed by Professors Chen C for bibliometric analysis and visualization (17, 30). In our study, CiteSpace was applied to map the dual-map overlay of journals and to analyze reference with Citation Bursts.

The R package “bibliometrix” (version 3.2.1) (https://www.bibliometrix.org) was applied for a thematic evolution analysis and to construct a global distribution network of publications of exosomes in AIDs (32). The quartile and impact factor of the journal are obtained from Journal Citation Reports 2020. Additionally, Microsoft Office Excel 2019 was used to conduct quantitative analysis of publication.

## Results

### Quantitative Analysis of Publication

**Figure 2:**
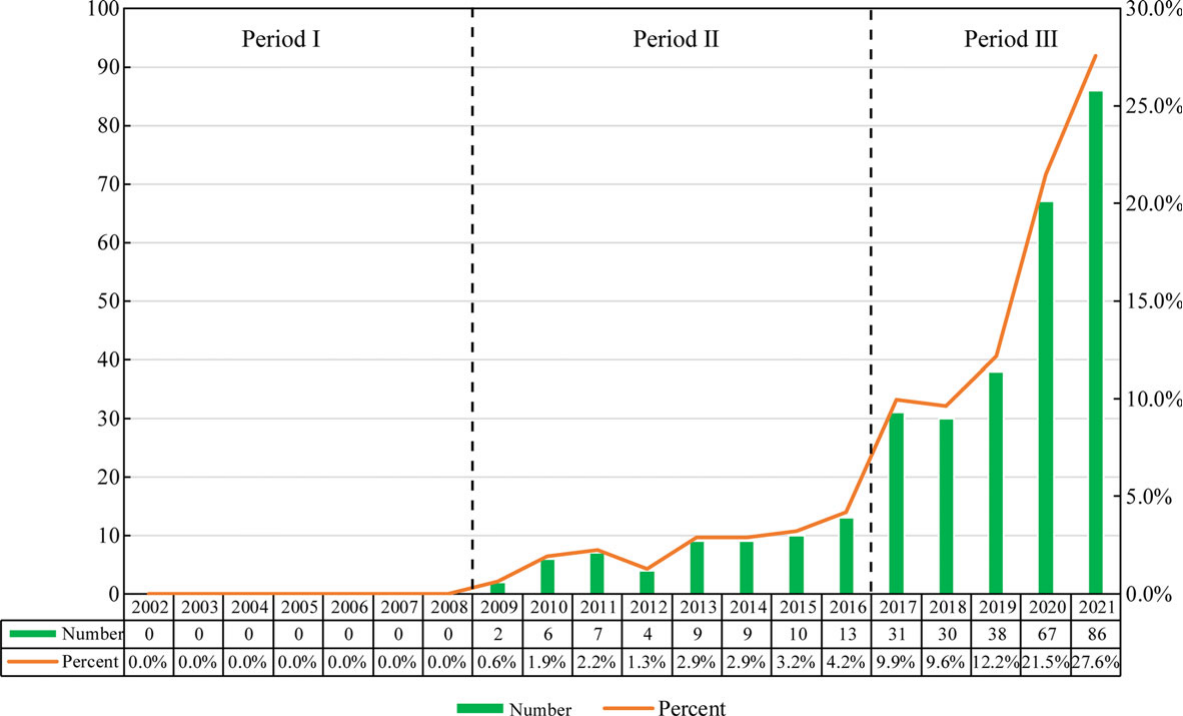
Annual output of research of exosomes in AIDs.

According to our search strategy, there were a total of 312 studies of exosomes in AIDs in the past two decades, including 157 “articles” and 155 “reviews”. Judging from the growth rate of the number of publications each year, the whole period can be divided into three parts: Period I (2002-2008), Period II (2009-2016) and Phase III (2017-2021). As shown in **Figure 2**, the number of publications in Phase I is 0, and the research of exosomes in AIDs has not been conducted during this period. The number of publications in Phase II is relatively small, with an average annual publication number of about 7.5, which is in the initial stage of the research of exosomes in AIDs. The number of publications in Phase III began to increase significantly, with an average annual number of about 50.4. The number of relevant publications published in 2017 was 31, 2.4 times that of 2016. In 2021, the number of publications of exosomes in AIDs reached 86. In the past five years (Phase III), the number of publications of exosomes in AIDs has shown an upward trend year by year, and the total number of papers in this stage has increased significantly compared with the other two stages.

### Country and Institutional Analysis

**Figure 3:**
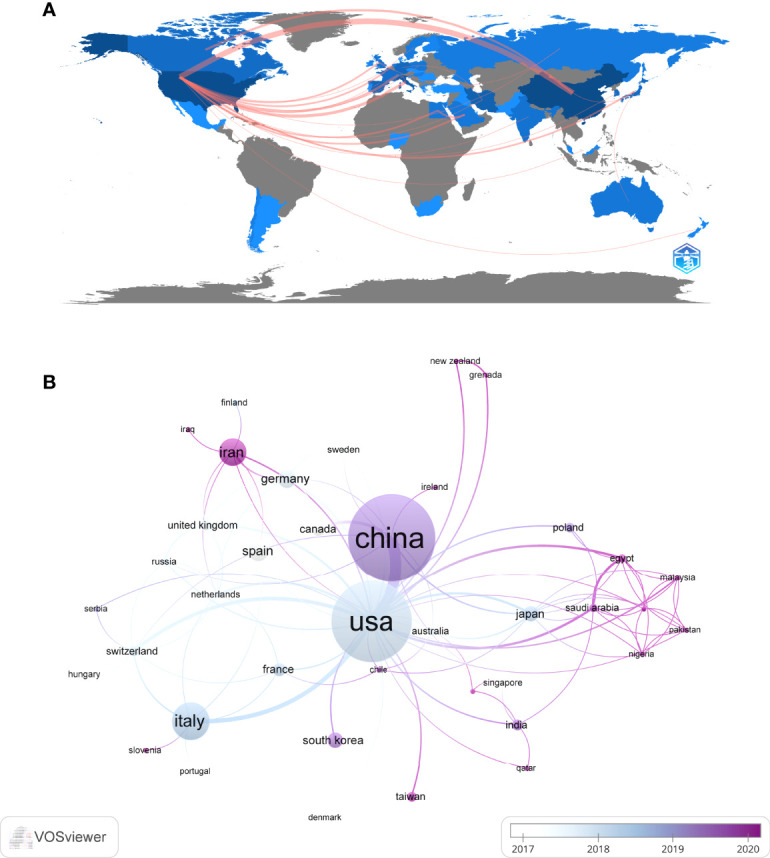
The geographical distribution **(A)** and visualization of countries **(B)** on research of exosomes in AIDs.

These publications came from 48 countries and 122 institutions. The top ten countries are distributed in Asia, North America, and Europe, mainly in Asia (n=4) and Europe (n=4) (**Table 1**). Among the countries, the country with the largest number of publications is China (n=97, 23.3%), followed by The United States (n=89, 21.4%), Italy (n=34, 8.2%), Iran (n=23, 5.5%). The combined number of publications from China and the United States accounted for almost half of the total (44.7%). Subsequently, we filtered and visualized 48 countries based on the number of publications more than or equal to 2, and constructed a collaborative network based on the number and relationship of publications in each country (**Figure 3**). Notably, there is a lot of active cooperation between different countries. For example, China has close cooperation with the United States, Canada, and Japan; the United States has active cooperation with Italy, Switzerland, Germany, and Iran.

**Table 1:** Top 10 countries and institutions on research of exosomes in AIDs.

Rank	Country	Counts	Institution	Counts
1	China (Asia)	97(23.3%)	Central South University (China)	7(1.7%)
2	The United States (North America)	89(21.4%)	Sun Yat Sen University (China)	7(1.7%)
3	Italy (Europe)	34(8.2%)	Tianjin Medical University (China)	7(1.7%)
4	Iran (Asia)	23(5.5%)	University of Pennsylvania (The United States)	7(1.7%)
5	Spain (Europe)	17(4.1%)	Jiangsu University (China)	6(1.4%)
6	Germany (Europe)	14(3.4%)	Shahid Beheshti University Medical Sciences (Iran)	6(1.4%)
7	Japan (Asia)	11(2.6%)	Tehran University of Medical Sciences (Iran)	6(1.4%)
8	South Korea (Asia)	11(2.6%)	China Medical University (China)	5(1.2%)
9	Canada (North America)	10(2.4%)	Nih National Institute of Dental Craniofacial Research Nidcr (The United States)	5(1.2%)
10	France (Europe)	9(2.2%)	Shandong University (China)	5(1.2%)

**Figure 4:**
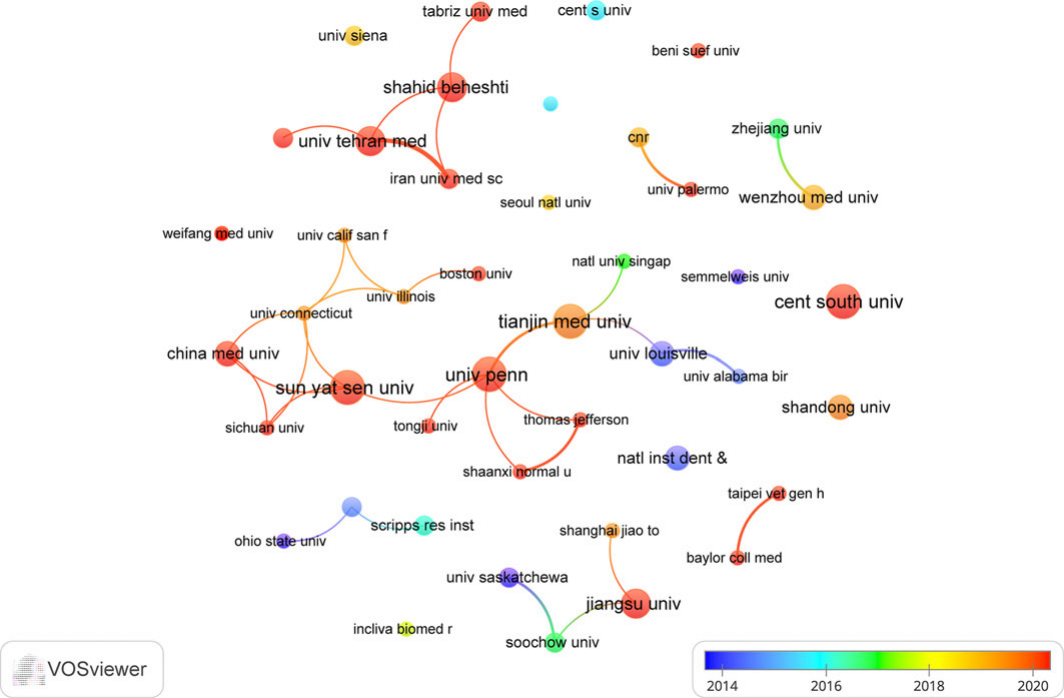
The visualization of institutions on research of exosomes in AIDs.

The top 10 institutions are located in 3 countries, with three-fifths of them located in China. The four institutions that published the most relevant papers are: Central South University (n=7, 1.7%), Sun Yat Sen University (n=7, 1.7%), Tianjin Medical University (n=7, 1.7%) and University of Pennsylvania (n=7, 1.7%). Subsequently, we selected 44 institutions based on the minimum number of publications equal to 3 for visualization, and constructed a collaborative network based on the number and relationship of publications of each institution (**Figure 4**). As shown in **Figure 4**, the cooperation between Sun Yat Sen University, Tianjin Medical University and University of Pennsylvania is very close, and there is active cooperation between Shahid Beheshti University Medical Sciences, Tehran University of Medical Sciences, Iran University of Medical Science. In addition, we note that Central South University, although it publishes the most papers, has no partnership with other institutions.

### Journals and Co-Cited Journals

**Figure 5:**
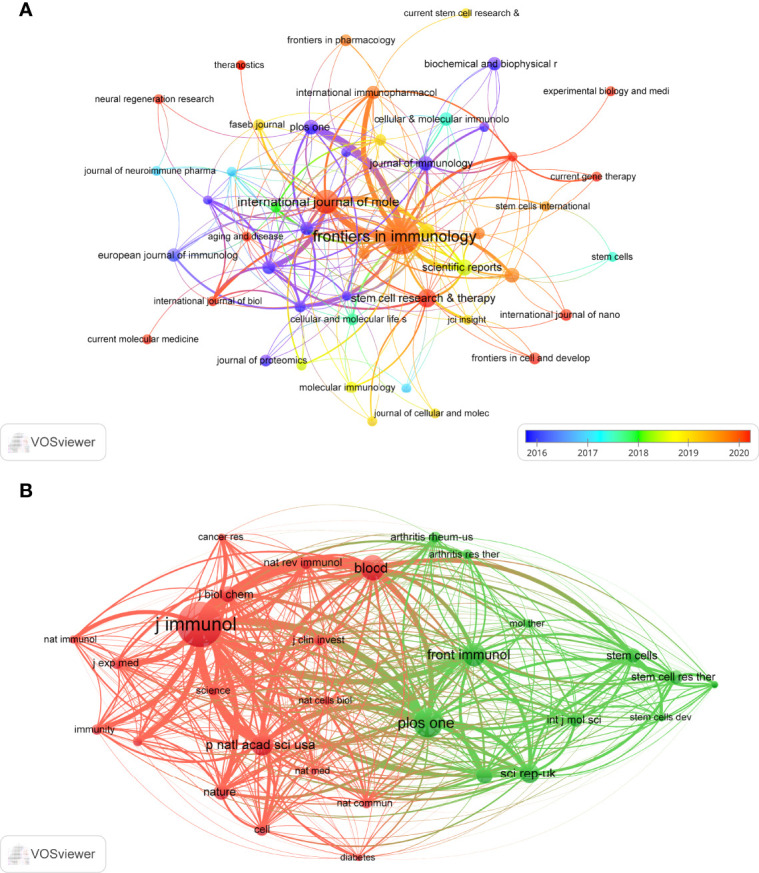
The visualization of journals **(A)** and co-cited journals **(B)** on research of exosomes in AIDs.

Publications related to exosomes in AIDs were published in 169 journals. *Frontiers in Immunology* published most papers (n=38, 12.2%), followed by *International Journal of Molecular Sciences* (n=16, 5.1%), *Stem Cell Research & Therapy* (n=8, 2.6%) and *Scientific Reports* (n=6, 1.9%). Among the top 15 journals, the journal with the highest impact factor is *Cellular & Molecular Immunology* (IF=11.53), followed by *Molecular Therapy* (IF=11.46). Subsequently, we screened 48 journals based on the minimum number of relevant publications equal to 2 and mapped the journal network (**Figure 5A**). **Figure 5A** shows that *Frontiers in Immunology* has active citation relationships with *International Journals of Molecular Sciences*, *International Immunopharmacology*, and *Plos One*, etc.

As shown in **Table 2**, among the top 15 co-cited journals, 4 journals were cited more than 500 times, and *Journal of Immunology* (Co-citation=1005) was the most cited journal, followed by *International Journal of Molecular Sciences* (Co-citation=651), *Blood* (Co-citation=568) and *Frontiers in Immunology* (Co-citation=514). In addition, the impact factor of *Nature reviews Immunology* is the highest (IF=53.11), followed by *Nature* (IF=49.93). Journals with the minimum co-citation equal to 150 were filtered to map the co-citation network (**Figure 5B**). As shown in **Figure 5B**, *Journal of immunology* has positive co-citation relationships with *Blood*, *Plos one*, *Frontiers in immunology*, etc.

**Table 2:** Top 15 journals and co-cited journals for research of exosomes in AIDs.

Rank	Journal	Count	IF	Q	Co-cited Journal	Co-citation	IF	Q
1	Frontiers in immunology	38 (12.2%)	7.56	Q1	Journal of Immunology	1005	5.42	Q2
2	International Journal of Molecular Sciences	16 (5.1%)	5.92	Q1	Plos One	651	3.24	Q2
3	Stem Cell Research & Therapy	8 (2.6%)	6.83	Q1	Blood	568	23.63	Q1
4	Scientific Reports	6 (1.9%)	4.38	Q1	Frontiers in Immunology	514	7.561	Q1
5	Autoimmunity Reviews	5 (1.6%)	9.75	Q1	Proceedings of the National Academy of Sciences of the United States of America	494	9.58	Q1
6	Journal of Cellular Physiology	5 (1.6%)	6.38	Q1	Scientific reports	451	4.38	Q1
7	Journal of Immunology	5 (1.6%)	5.42	Q2	Journal of extracellular vesicles	370	25.85	Q1
8	Plos One	5 (1.6%)	3.24	Q2	The Journal of biological chemistry	350	5.157	Q2
9	Biochemical and Biophysical Research Communications	4 (1.3%)	3.58	Q2	Nature	343	49.93	Q1
10	Cellular & Molecular Immunology	4 (1.3%)	11.53	Q1	Stem cells	317	6.28	Q1
11	European Journal of Immunology	4 (1.3%)	5.53	Q2	Stem Cell Research & Therapy	295	6.83	Q1
12	International Immunopharmacology	4 (1.3%)	4.94	Q2	International journal of molecular sciences	289	5.92	Q1
13	Journal of Neuroimmunology	4 (1.3%)	3.48	Q3	Nature reviews Immunology	282	53.11	Q1
14	Molecular Therapy	4 (1.3%)	11.46	Q1	Cell	272	41.59	Q1
15	Cells	3 (1%)	6.6	Q2	The Journal of experimental medicine	263	14.31	Q1

**Figure 6:**
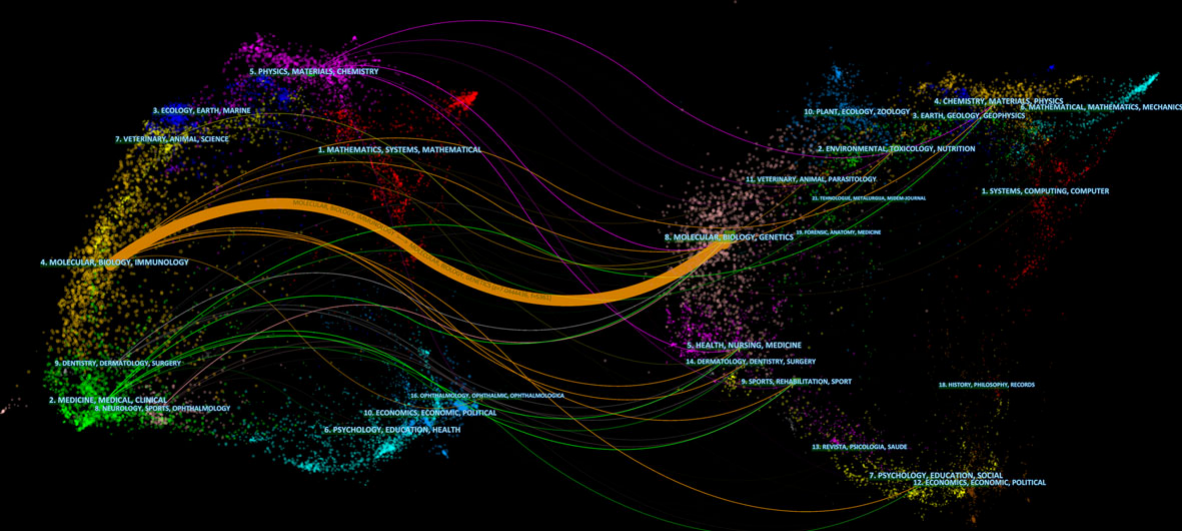
The dual-map overlay of journals on research of exosomes in AIDs.

The dual-map overlay of journals shows the citation relationships between journals and co-cited journals, with clusters of citing journals on the left and clusters of cited journals on the right (33). As shown in **Figure 6**, the orange path is the main citation path, which represents the research published in Molecular/Biology/Genetics journals is mainly cited by literature in Molecular/Biology/Immunology journals.

### Authors and Co-Cited Authors

**Figure 7:**
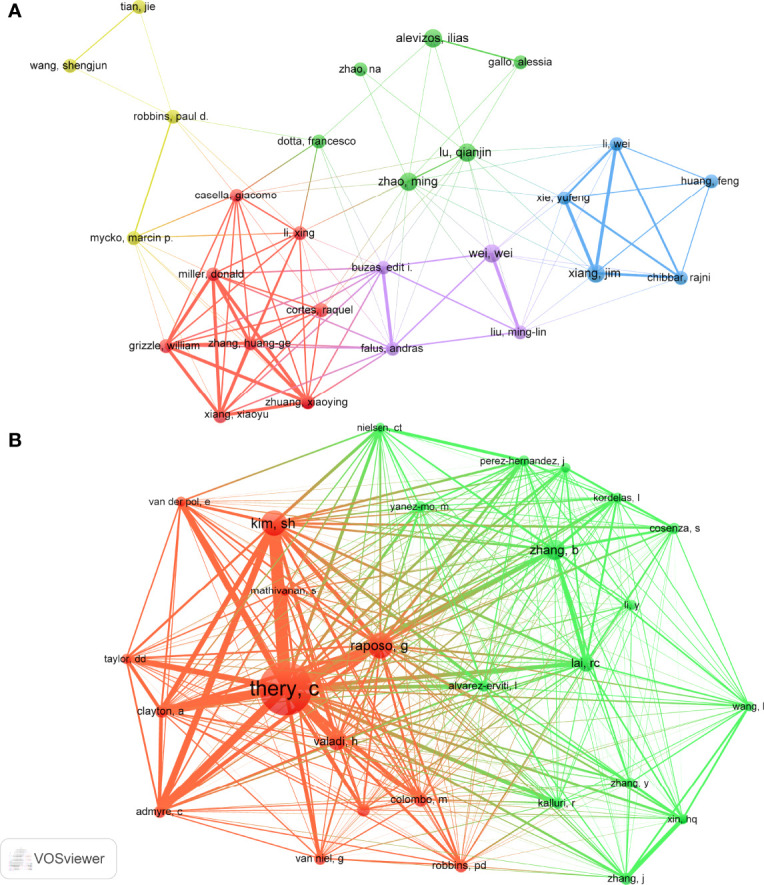
The visualization of authors **(A)** and co-cited Authors **(B)** on research of exosomes in AIDs.

A total of 1858 authors participated in research of exosomes in AIDs. Among the top 10 authors, five authors each published 4 papers (**Table 3**). We build a collaborative network based on authors whose number of published papers is more than or equal to 3 (**Figure 7A**). Ilias Alevzos, Qianjin Lu, Wei Wei, Jim Xiang and Ming Zhao have the largest nodes because they publish the most related publications. Besides, we observed close collaboration among multiple authors. For example, Xiaoyu Xiang has close cooperation with Xiaoying Zhuang, Huang-Ge Zhang and William Grizzle; Jim Xiang has active cooperation with Yufeng Xie, etc.

**Table 3:** Top 10 authors and co-cited authors on research of exosomes in AIDs.

Rank	authors	count	Co-Cited Authors	citations
1	Ilias Alevizos	4	Clotilde Théry	223
2	Qianjin Lu	4	Graça Raposo	87
3	Wei Wei	4	Seon Hee Kim	85
4	Jim Xiang	4	Bo Zhang	68
5	Ming Zhao	4	Hadi Valadi	58
6	Edit I Buzás	3	Ruenn Chai Lai	55
7	Giacomo Casella	3	Marina Colombo	45
8	Minghan Chen	3	Aled Clayton	43
9	Rajni Chibbar	3	Paul D Robbins	42
10	Raquel Cortes	3	Guillaume van Niel	42

Among the 15815 co-cited authors, 6 authors were co-cited more than 50 times (**Table 3**). The most co-cited author is Clotilde Théry (n=223), followed by Graça Raposo (n=87) and Seon Hee Kim (n=85). Authors with minimum co-citations equal to 30 were filtered to map co-citation network graphs (**Figure 7B**). As shown in **Figure 7B**, there are also active collaborations among different co-cited authors, such as Clotilde Théry and Seon Hee Kim, Bo Zhang and Ruenn Chai Lai.

### Co-Cited References

**Figure 8:**
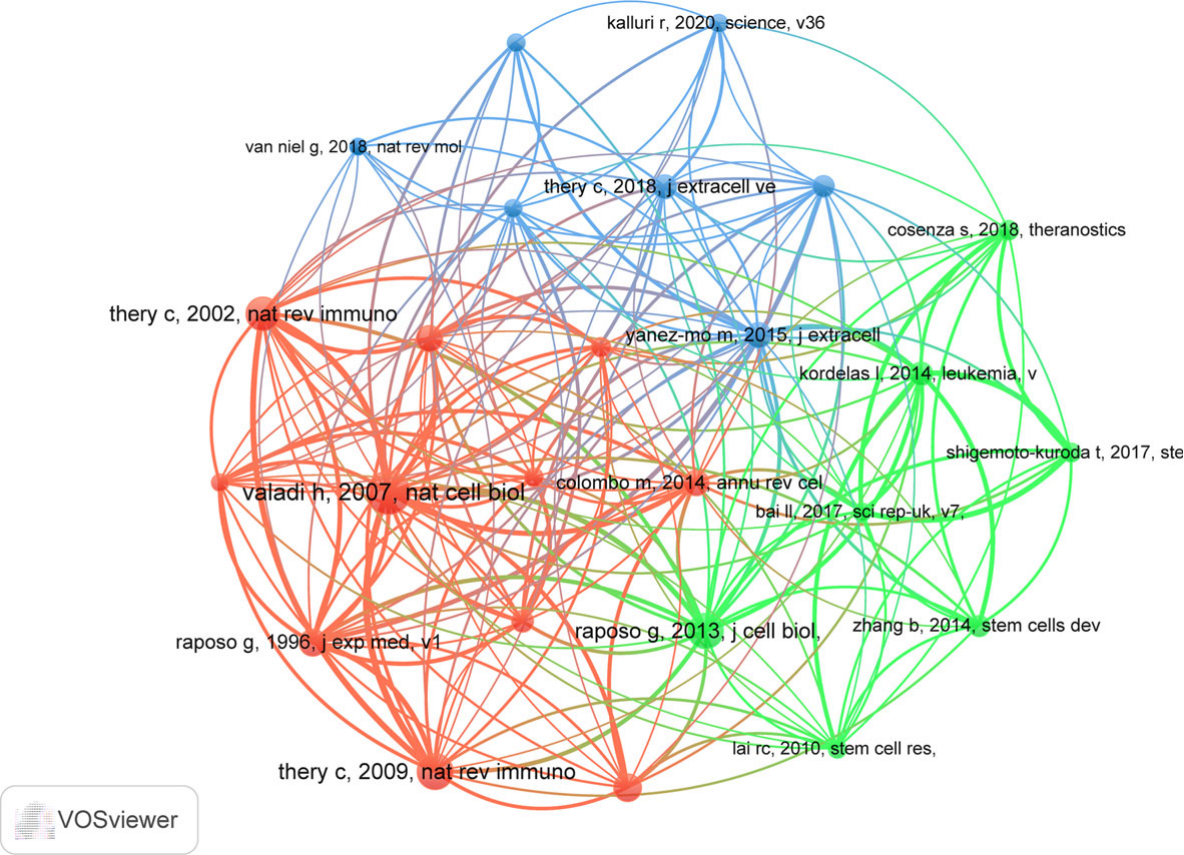
The visualization of co-cited references on research of exosomes in AIDs.

There are 20,422 co-cited references on research of exosomes in AIDs over the past two decades. In top 10 co-cited references (**Table 4**), all references were co-cited at least 32 times, and a reference was co-cited more than 50 times (34). We selected references with co-citation more than or equal to 23 for the construction of the co-citation network map (**Figure 8**). According to **Figure 8**, “Valadi H, 2007, Nat Cell Biol” shows active co-cited relationships with “Thery C, 2009, Nat Rev Immunol”, “Thery C, 2002, Nat Rev Immunol” and “Raposo G, 1996, J Exp Med”, etc.

**Table 4:** Top 10 co-cited references on research of exosomes in AIDs.

Rank	Co-cited reference	Citations
1	Valadi H, 2007, Nat Cell Biol, V9, P654 (34)	58
2	Thery C, 2009, Nat Rev Immunol, V9, P581 (35)	48
3	Raposo G, 2013, J Cell Biol, V200, P373 (36)	47
4	Thery C, 2002, Nat Rev Immunol, V2, P569 (37)	45
5	Thery C, 2006, Curr Protoc Cell Biol, Vchapter 3 (38)	37
6	Raposo G, 1996, J Exp Med, V183, P1161 (39)	36
7	Alvarez-erviti L, 2011, Nat Biotechnol, V29, P341 (40)	35
8	Colombo M, 2014, Annu Rev Cell Dev Bi, V30, P255 (41)	34
9	Yanez-mo M, 2015, J Extracell Vesicles, V4 (42)	34
10	Thery C, 2018, J Extracell Vesicles, V7 (43)	32

### Reference with Citation Bursts

**Figure 9:**
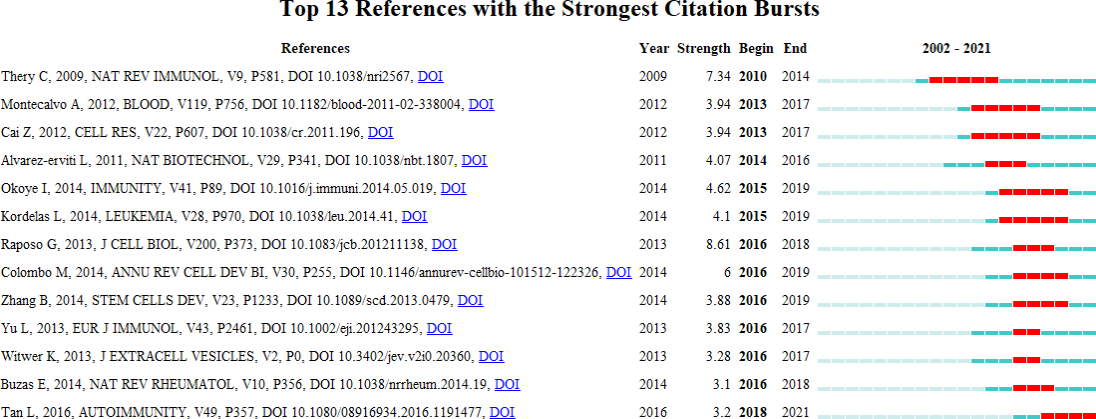
Top 13 references with strong citation bursts. A red bar indicates high citations in that year.

Reference with citation bursts refers to those references that are frequently cited by scholars in a certain field over a period of time. In our study, 13 references with strong citation bursts were identified by CiteSpace (**Figure 9**). As shown in **Figure 9**, every bar represents a year, and the red bar represents strong citation burstiness (44). Citation bursts for references appeared as early as 2010 and as late as 2018. The reference with the strongest citation burst (strength=8.61) was titled “Extracellular vesicles: exosomes, microvesicles, and friends”, authored by Graça Raposo et al. with citation bursts from 2016 to 2018. The reference with the second strongest citation burst (strength=7.34) was titled “Membrane vesicles as conveyors of immune responses “, published in *Nature reviews Immunology* by Clotilde Théry et al. with citation bursts from 2010 to 2014. Overall, the bursts strength of these 13 references ranged from 3.1 to 8.61, and endurance strength was from 2 to 5 years. **Table 5** summarizes the main research contents of the 13 references in the order of the literature in **Figure 9**.

**Table 5:** The main research contents of the 13 references with strong citations bursts.

Rank	Strength	Main research content
1	7.34	The role of exosomes in the communication between different cells, such as immune and tumor cells (35).
2	3.94	Mechanism of miRNA delivery by dendritic cell-derived exosomes (45).
3	3.94	Dendritic cell-derived exosomes induce generation of regulatory T cells to alleviate inflammatory bowel disease (46).
4	4.07	Targeted exosomes can deliver siRNA to mouse brain for therapeutic effect (40).
5	4.62	Regulatory T cell-derived exosomes can inhibit the proliferation and secretion of inflammatory mediators of Pathogenic Th1 Cells by delivering miR-Let-7d (47).
6	4.1	MSC-derived exosomes could treat graft-versus-host disease (48).
7	8.61	The characterization of exosomes and mechanisms of their generation, secretion, and effect (36).
8	6	The formation, delivery, and intercellular communication of exosomes (41).
9	3.88	Infusion of MSC exosomes elevates Tregs levels and increases survival in allogenic skin graft mice (49).
10	3.83	Genetically modified dendritic cell-derived exosomes deliver TGF-β1 and maintain the regulatory capacity of Treg cells to suppress EAE in mice (50).
11	3.28	The standardization of sample preparation, isolation, purification and subsequent analysis in EVs research (51).
12	3.1	The development and prospect of Exosomes for prevention and treatment of AIDs (52).
13	3.2	The advances of exosomes in the pathogenesis and therapeutics of AIDs (53).

### Hotspots and Frontiers

Through the co-occurrence analysis of keywords, we could quickly capture research hotspots in a certain field. **Table 6** shows the top 20 high-frequency keywords in research of exosomes in AIDs. Among these keywords, mesenchymal stem cells and miRNA appeared more than 40 times, which represented the main research direction of exosomes in AIDs.

**Table 6:** Top 20 keywords on research of exosomes in AIDs.

Rank	Keywords	Counts	Rank	Keywords	Counts
1	exosomes	149	11	inflammation	18
2	extracellular vesicles	63	12	therapy	15
3	mesenchymal stem cells	48	13	microvesicles	14
4	microRNAs	41	14	immunomodulation	12
5	autoimmune diseases	31	15	cancer	10
6	autoimmunity	27	16	mesenchymal stromal cells	9
7	multiple sclerosis	23	17	experimental autoimmune encephalomyelitis	8
8	biomarkers	22	18	proteomics	8
9	rheumatoid arthritis	21	19	sjogren’s syndrome	8
10	systemic lupus erythematosus	19	20	dendritic cells	7

**Figure 10:**
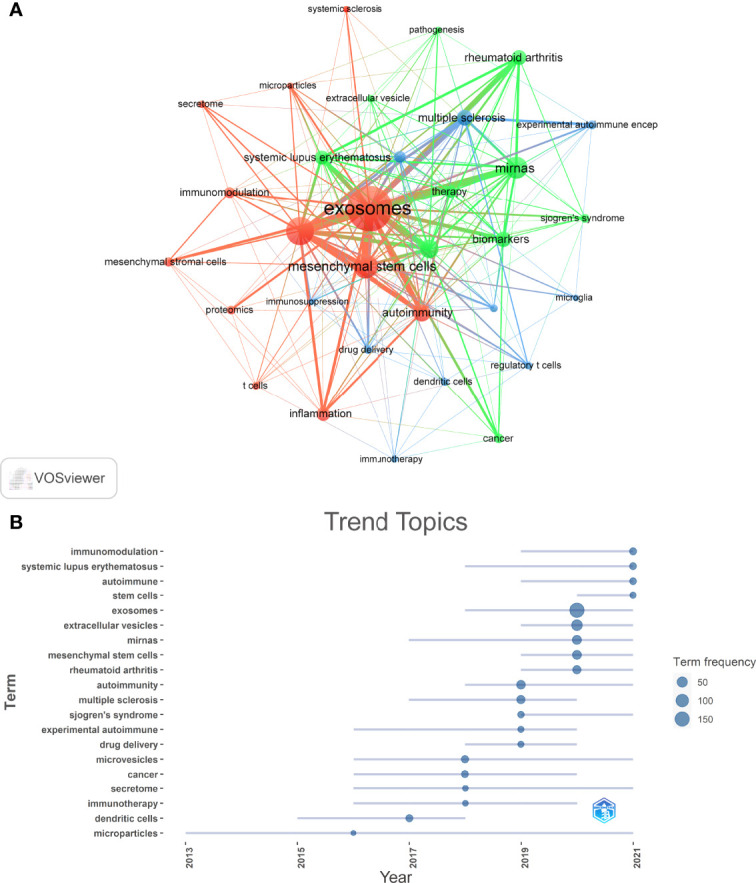
Keyword cluster analysis **(A)** and trend topic analysis **(B)**.

We filtered keywords with the number of occurrences more than or equal to 4 and performed cluster analysis through VOSviewer (**Figure 10A**). The thicker the lines between the nodes, the stronger the connection between the keywords. As shown in **Figure 10A**, we obtained three clusters in total, representing three research directions. The keywords in green clusters consists of microRNAs, biomarkers, autoimmune diseases, rheumatoid arthritis, systemic lupus erythematosus, etc. The keywords in red clusters consist of MSCs, immune regulation, inflammation, autoimmunity, etc. The keywords in blue clusters consist of dendritic cells, regulatory T cells, drug delivery, multiple sclerosis, etc. The trend topic analysis of the keywords (**Figure 10B**) showed that from 2002 to 2016, the research in this period mainly focused on microvesicles and microparticles, and that the main keywords were microvesicles, immunotherapy, secretome, dendritic cells, microparticles. Since 2017, Scholars have begun to actively explore the pathogenesis and therapeutic potential of exosomes in AIDs, and the main keywords are mesenchymal stem cells, immunomodulation, microRNA, drug delivery, inflammation, rheumatoid arthritis, etc. Besides, these three keywords: mesenchymal stem cells, immunomodulation, and microRNA have appeared frequently in the past two years (2020-2021), so they are very likely to represent the current research hotspots of exosomes in AIDs.

## Discussion

### General Information

The annual publications from 2002 to 2008 is 0, indicating that the research of exosomes in AIDs has not been carried out at this time, and that the research foundation between exosomes and AIDs is lacking. From 2009 to 2016, research in this field was still in its infancy, with an average annual publication of 7.5 papers. From 2017 to 2021, the number of publications began to increase significantly, with an average annual publication of 50.4 papers. The number of related publications has grown rapidly over the past five years, which indicates the research of exosomes in AIDs is being in an explosive period, and that related research has attracted more and more scholars’ attention.

China and the United States are major countries conducting research of exosomes in AIDs, and China ranks first. About 60% of the top 10 research institutions are located in China, followed by the United States (n=2, 20%) and Iran (n=2, 20%). We noticed the close cooperation among four countries: the United States, China, Italy, and Iran. In addition, Iran has active collaborations with the United States, Spain and Germany. When it comes to research institutions, there is a good cooperative relationship between some of them, such as Sun Yat Sen University, Tianjin Medical University and University of Pennsylvania. However, we also found Central South University, while publishing the most papers, had little collaboration with other institutions, which will be detrimental to the long-term development of academic research. Although there are cooperative relations between some countries, the breadth and intensity of cooperation between institutions are not ideal. For example, there is only a small amount of cooperation between institutions in the United States and China. Clearly, this situation will hinder the development of the research field in the long run. Therefore, we strongly recommend research institutions in various countries carry out extensive cooperation and communication to jointly promote the development of exosomes in AIDs.

Most of the research of exosomes in AIDs was published in *Frontier in immunology* (IF=7.561, Q1), indicating it is currently the most popular journal in this research field. Among the journals, the journal with the highest impact factor is *Cellular & Molecular Immunology* (IF=11.53, Q1), followed by *Molecular Therapy* (IF=11.46, Q1). Regarding the co-cited journals, we could find most of them are high-impact Q1 journals. Obviously, these journals are high-quality international journals, providing support for the study of exosomes in AIDs. What’s more, the current research of exosomes in AIDs is mainly published in Molecular, Biology, and Immunology related journals, and very few studies are published in clinically related journals, indicating that most of the current research is still in the stage of basic research.

From the perspective of the author, Ilias Alevzos, Qianjin Lu, Wei Wei, Jim Xiang and Ming Zhao published the most articles, with 4 papers per capita. Professor Ilias Alevzos published 4 papers, two of which pointed out most of the human miRNAs are present in serum and saliva-derived exosomes, and that these miRNAs could be diagnostic markers for related diseases (54, 55). They also found exosomes derived from T cells and B cells can transmit miR-142-3p and miR-BART13-3p to the epithelial cells, thereby impairing the function of epithelial cells and promoting the occurrence of SS (56, 57). Qianjin Lu and Ming Zhao co-published 4 papers, two of which reviewed the research progress of exosomes in the pathogenesis, immune regulation and treatment of AIDs (53, 58), and the two other articles pointed out the mechanism of serum exosomal miR-451a and miR-642-3p involved in the pathogenesis of SLE and SS (59, 60). Wei Wei published 4 papers, three of which reviewed the pathogenesis of extracellular vesicles in vasculitis and lupus nephritis (LN) (61–63), and another article pointed out plasma exosomes participate in the inflammation process of microscopic polyangiitis (64). Besides, four papers authored by Jim Xiang mainly studied the immunomodulatory effects of exosomes derived from CD4^+^ T cells, CD8^+^25^+^ regulatory T cells (65–67) and dendritic cells (68) in mice. In general, the above studies mainly focus on the pathogenesis, diagnosis and treatment of exosomes in AIDs.

In terms of co-cited authors, Théry C (citation=223) is the most frequently cited author, followed by Raposo G (citation=87) and Kim SH (citation=85). In 2001, Théry C conducted a detailed identification of the protein components in dendritic cell-derived exosomes, and found that the components in exosomes were significantly different from those of apoptotic vesicles (69). The next year, Professor Théry C found that dendritic cell-derived exosomes could indirectly activate CD4^+^ T cells and amplify the initiation of primary adaptive immune responses (70), and this paper laid the foundation for the study of dendritic cells in AIDs (46, 68). In the same year, a review published by Théry C in *Nature reviews Immunology* summarized the biological effects of exosomes on the immune system and discussed the potential roles of secreted vesicles as intercellular messengers (37). Subsequently, a study published in 2006 summarized the approaches for purifying exosomes from different sources, and proposed a protocol for evaluating the purity and homogeneity of exosomes (38). In recent years, a paper published by Théry C and his colleagues summarizes important details for the separation/enrichment, classification and identification of EVs which has a standardized and instructive impact on the current research of exosomes in various fields (43). Obviously, the achievements of Théry C have laid a theoretical and experimental foundation for research of exosomes in AIDs.

### Knowledge Base

A co-cited reference refers to a reference that is cited together by multiple other publications, so that co-cited references could be considered as the research basis in a field (71). In this bibliometric study, we selected the 10 co-cited references with the highest number of co-citations to determine the research basis of exosomes in AIDs. Hadi Valadi et al. published the most co-cited study in 2007, and this study firstly points out that exosomes can communicate with other cells by transmitting mRNA and miRNA, which lays the foundation for the study of mechanism in exosomes (34). So far, miRNAs are still the research hotspot of exosomes in AIDs. Professor Clotilde Théry has published 4 papers in these 10 total cited papers, and these four papers have been discussed in the previous paragraph, so they will not be repeated here. Graça Raposo published 2 papers in these 10 co-cited papers, the first of which was published in *The Journal of experimental medicine* in 1996, which first proposed that EB virus-transfected B cells can perform antigen presentation by secreting exosomes, which stimulated people’s interest in the role of exosomes in antigen presentation (39). The second study summarizes new progress of EVs in their formation, targeting, and function (36). In 2011, Lydia Alvarez-Erviti et al. published the 7th co-cited paper in *Nature biotechnology* (40). They found exosomes delivered siRNAs to the mouse brain in a targeted manner and cause therapeutic effects, which provided a new idea for engineering exosomes to treat diseases (40). In 2014, the 8th co-cited reference authored by Marina Colombo et al. outlined the formation, delivery, and intercellular communication of exosomes (41). The following year, the last co-cited paper authored by María et al. summarizes the contents and functions of EVs, and the physiological mechanisms of EVs (42). Overall, the top 10 co-cited references focus on the following topics: biological function of exosomes, identification and purification of exosomes, components of exosomes and targeted delivery, which are the research basis in exosomes.

### Hotspots and Frontiers

References with citation bursts represent emerging topics within a particular research field, as these references have been frequently cited by researchers in recent years (72). According to the main research contents of references with strong citations bursts (**Table 5**), we can find studying the biological role and pathogenesis of endogenous exosomes in AIDs and how to use exogenous exosomes to treat AIDs are the current major topics in the research of exosomes in AIDs. These exogenous exosomes include exosomes derived from cells with immunomodulatory effects (such as MSCs, dendritic cells and Treg) or engineered exosomes (such as exosomes loaded with siRNA and TGF-β, etc.).

In addition to references with citation bursts, keywords can also help us quickly capture the distribution and evolution of hotspots in the research field of exosomes in AIDs. Excluding keywords such as exosomes, extracellular vesicles, and AIDs, **Table 6** mainly includes the following keywords: mesenchymal stem cells, microRNA, biomarkers, treatment, and immunomodulation. According to keyword clustering analysis and trend topic analysis (**Figure 10**), we concluded that the research of exosomes in AIDs mainly focuses on the following aspects:

#### MSC-Exos

Among the exosomes-producing parent cells, MSCs have attracted the attention of many researchers (4). MSCs have powerful immune regulation and tissue repair effects, which are easily obtained from umbilical cord, bone marrow, blood, and adipose tissue, etc (6). In recent years, MSCs have gradually been used in many clinical trials to treat AIDs such as RA, T1D, MS, and SLE, and most of the results show that MSCs have good therapeutic effect and safety on AIDs (73). In these clinical trials, most adverse events were mild. For example, in a clinical trial using MSCs to treat RA (N=53), 94% of adverse events were mild events such as fever, respiratory infections, and headache (74). However, several animal experiments have shown that MSCs may increase the risk of cancer by inhibiting the function of CD8^+^ T cells (75–77). Besides, most of the relevant clinical trials are not unblinded, and the number of patients included is small (N<110) (78). There is currently no large multicenter placebo-controlled trials, so the efficacy and safety of MSCs in the treatment of AIDs needs to be further confirmed (78). MSC-derived exosomes (MSC-Exos) have biological functions similar to MSCs, but MSC-Exos are more stable and less toxic, which can largely avoid the body’s triggering immune response and capillary embolism and other adverse events, so researchers are actively exploring the strategy of MSCs-Exos in the treatment of AIDs (4, 10, 79).

**Figure 11:**
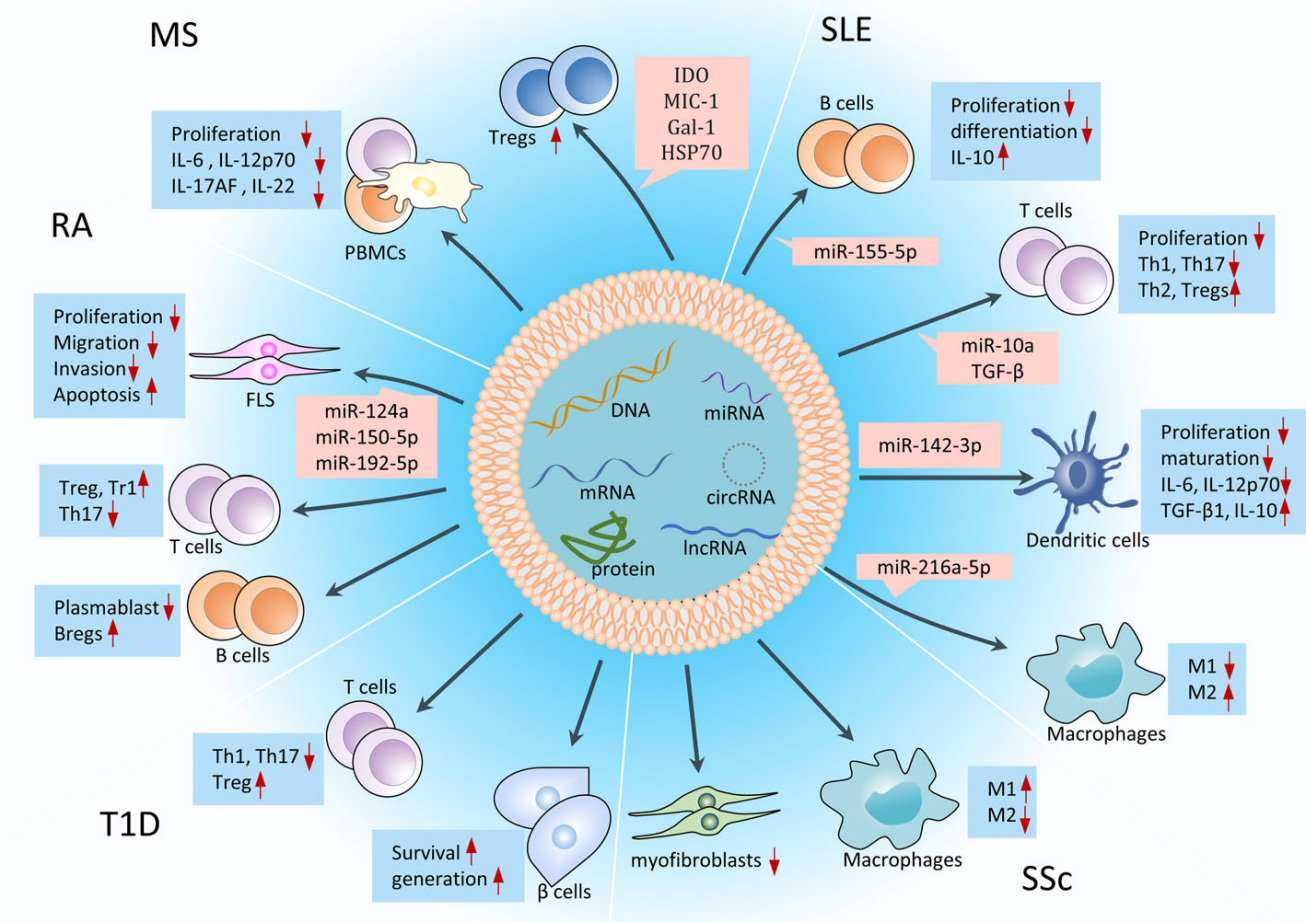
Immunomodulation and tissue repair effects of MSC-Exos in AIDs. MSC-Exos can differentiate immune cells in the direction of suppressing inflammation, reduce the secretion of inflammatory cytokines, and repair damaged tissue cells in the body, such as FLS and β cells. miRNAs are the most studied substances on the mechanisms associated with MSC-Exos in the treatment of AIDs. Tr1, T regulatory type 1 cells; Th 17, T helper 17 effector cells; miR, microRNA; IDO, indoleamine 2,3-dioxygenase; MIC-1, macrophage inhibitory cytokine 1; Gal, galectin-1; HSP70, heat shock protein 70.

Compared with single-factor drugs (traditional DEMARDs drugs, small-molecule drugs, and biologics), MSC-Exos are complex mixtures of factors targeting different therapeutic pathways, so their immunomodulatory and tissue repair effects are much stronger (**Figure 11**). In RA, MSC-Exos could deliver miR-150-5p to fibroblast-like synoviocytes (FLS) and reduce the production of matrix metalloproteinase 14 and vascular endothelial growth factor to reduce invasion and migration of FLS and ultimately treat RA. MSC-Exos also inhibited the invasion and migration of MH7A cells (RA-FLS cell lines) by delivering miR-124a (80). Besides, MSC-Exos can inhibit the formation of Th17 and plasmablasts and promote the formation of Treg cells in CIA mice (4). In SLE, MSC-Exos can deliver miR-155-5p, miR-10a, miR-142-3p and miR-216a-5p to produce anti-inflammatory immunosuppressive effects on immune cells such as B cells and T cells (81). Riazifar et al. (82) found that MSC-Exos contained several mRNAs (such as indoleamine 2,3-dioxygenase, thymosin beta 10 pseudogene 1, etc.) and proteins (such as macrophage inhibitory cytokine 1, galectin-1, heat shock protein 70, etc.) with anti-inflammatory properties, and these anti-inflammatory substances could promote the formation of Tregs in the mouse spinal cord and inhibit the secretion of IL-6, IL-12p70, IL-17AF and IL-22 from PBMCs to cure MS. However, the therapeutic mechanism, such as how indoleamine 2,3-dioxygenase in Exos promotes the formation of Tregs, remains to be further studied. Co-culture experiments also showed that MSC-Exos can inhibit the proliferation of human PBMCs (83), whether it has a similar therapeutic effect in MS patients remains to be further studied. In SSc, MSC-Exos can exert antifibrotic and anti-inflammatory effects by promoting M1 macrophage polarization and inhibiting M2 macrophage polarization (84). In T1D, MSC-Exos are able to increase the ratio of Treg in PBMCs of T1D patients and inhibit the proliferation of Th1 and Th17 cells in T1D mice (85). In addition, MSC-Exos have a positive effect on promoting the survival and generation of β cells (85). Recent studies have shown that the content of protein in exosomes is much higher than that of miRNAs, so MSC-Exos are highly likely to treat AIDs mainly through proteins, which provides a new idea for the study of mechanism associated with MSC-Exos in the treatment of AIDs (7).

Although scholars have achieved good results in the use of MSC-Exos in the treatment of AIDs animal models, there is still a long way to go before its clinical application in the treatment of AIDs. Firstly, the manufacturing and preparation processes of MSC-Exos are tedious and complicated, which are still to be optimized. We should seriously consider standardized methods for MSC-Exos isolation, purification, quantification, and quality control, which can reduce batch effects and establish quality control for its clinical applications. Secondly, we can try to explore ways to increase the half-life of exosomes [such as MSC-Exos with an injectable hydrogel (86)] and determine which substances in MSC-Exos are mainly responsible for anti-inflammatory effects, and accordingly produce engineered exosomes that overexpress these substances to improve the efficacy of MSC-Exos. Finally, the route of administration, dose, infusion time, treatment interval, and long-term safety of MSC-Exos in the treatment of AIDs deserve further confirmation in more high-quality studies for its further clinical application in the future.

#### miRNA and Biomarkers

miRNAs are currently the most studied substances in the contents of exosomes (**Table 7**), and these endogenous exosomes mainly come from body fluids such as serum, plasma and urine of AIDs patients. On the one hand, miRNAs in endogenous exosomes in AIDs patients are of vital importance in promoting the onset of AIDs. For example, miR-548a-3p in serum exosomes of RA patients could inhibit the proliferation and activation of pTHP-1 cells and miR-6089 could regulated the generation of IL-6, IL-29, and TNF-α (87, 88). In SLE, let-7b and miR-21 in patients’ plasma exosomes can activate plasmacytoid DCs (pDCs) cells and allow them produce proinflammatory cytokines through the TLR7 signaling (98). In MS, let-7i in patients’ plasma exosomes can suppresses induction of Treg cells by targeting insulin like growth factor 1 receptor (IGF1R) and transforming growth factor beta receptor 1 (TGFBR1) (104). Cortes et al. found that miR-142-3p in T cell-derived exosomes could impair glandular cell function in SS by downregulating Calcium signaling and cAMP production (56). In a word, these endogenous exosomes can ultimately lead to the occurrence of AIDs by delivering miRNA to recipient cells and causing them to release pro-inflammatory factors or differentiate these cells in a pro-inflammatory direction.

**Table 7:** The contents of endogenous exosomes in the pathogenesis or diagnosis of AIDs.

Diseases	Origin of exosomes	Contents	Pathogenic mechanism or diagnostic value	references
RA	Serum of RA patients	miR-548a-3p	Inhibit the proliferation and activation of pTHP-1 cells	(87)
	Serum of RA patients	miR-6089	Regulated the generation of IL-6, IL-29, and TNF-α	(88)
	Serum of RA patients	miR-17	Destroy the Tregs homeostasis	(89)
	Serum of RA patients	miR-451a miR-25-3p	Early diagnosis of RA	(90)
	Serum of RA patients	amyloid A, LYVE-1	Associated with disease activity in RA	(91)
	FLS of CIA mice	miR-424	Enhance the inflammatory response of RA	(92)
	FLS of CIA mice	miR-106b	Suppresses chondrocyte proliferation and migration	(93)
	FLS of K/BxN mice	Id1	Induces angiogenesis	(94)
	PBMCs of CIA mice	LncRNA NEAT1	Promotes FLS viability and inflammation	(95)
SLE	Serum of SLE patients	miR-451a	Correlates with renal damage and intercellular communication role	(59)
	Serum of SLE patients	miR-21, miR-155	A potential diagnostic value for SLE and LN	(96)
	T cell of ECP-transgenic mice	ECP	Induction of interferon-γ and tissue inflammation	(97)
	Plasma of SLE patients	let-7b, miR-21	Activate pDC cells and allow them produce proinflammatory cytokines through the TLR7 signaling	(98)
LN	Urine of LN patients	miR-150	Increased profibrotic proteins in proximal tubular cells and podocytes	(99)
	Urine of LN patients and B6.MRLc1 mice	miR-26a	Regulates podocyte differentiation and cytoskeletal integrity	(100)
	Urine of LN patients	let-7a, miR-21	Guide the clinical stage of LN patients	(101)
	Urine of LN patients	miR-31, miR-107, miR-135b-5p	Early markers for predicting LN clinical response	(102)
	Urine of LN patients	miR-29c	Correlated with the degree of renal chronicity	(103)
MS	Plasma of MS patients	miR-let-7i	Suppress the induction of regulatory T cells	(104)
	Serum of MS patients	myelin basic protein, proteolipid protein, MOG	Enhance and/or perpetuate anti-myelin immune reactions	(105)
SSc	Serum of SSc patients	9 profibrotic miRNAs (such as let-7g-5p, miR-17–5p and miR-21–5p )	Induce the expression of profibrotic genes	(106)
T1D	Serum of T1D patients	miR-21-5p	Increased β cell apoptosis	(107)
	human Jurkat T cells	miR-142-3p, miR-142-5p, miR-155	Promote pancreatic β Cell death and may contribute to T1D development	(108)
MG	Plasma of MG patients	miR-106a-5p	Associated with MG severity	(109)
PsA	Plasma of MG patients	let-7b-5p, miR-30e-5p	Associated with the presence of PsA	(110)
SS	T cells of SS patients	miR-142-3p	Impairs glandular cell function through downregulating Calcium signaling and cAMP production in SS	(56)
HT	Serum of HT patients	TPO, HSP60, MHC-II	Causing DC activation *via* the NF-κB pathway, leading to an imbalance in CD4^+^ T lymphocyte differentiation	(111)
GD	Serum of GD patients	hsa_circRNA_000102	Involved in pathways of immune system activation, such as viral infection and interferon-beta signaling.	(112)

Many miRNAs can be biomarkers for disease diagnosis and prognosis. The results of miRNA microarray showed that the level of miR-548a-3p in serum exosomes of RA patients was significantly reduced, and was negatively correlated with inflammatory indicators such as serum CRP and rheumatoid factor (RF), so miR-548a-3p may serve as a biomarker for predicting disease activity in RA (87). In addition, miR451a (90), miR-25-3p (90), and miR-6089 (88) in serum exosomes also contribute to the diagnosis of early RA. Tan L et al. found that the level of miR-451a in serum exosomes of SLE patients was significantly reduced and correlated with disease activity, indicating that miR-451a could be a biomarker and therapeutic target for SLE (59). Besides, miR-150 (99), miR-29c (103) in the urinary exosomes of LN patients were associated with renal fibrosis, and miR-26a was associated with podocyte injury (100), so these three miRNAs may be biomarkers for renal fibrosis or podocyte injury in LN. Kimura K et al. found that the significantly elevated miR-let-7i in plasma exosomes of MS patients can promote the pathogenesis of MS by inhibiting the generation of regulatory T cells, indicating this miRNA is likely to become a potential biomarker of MS (104). Eight miRNAs in serum exosomes of SSc patients are associated with clinical symptoms of SSc patients, and these miRNAs are helpful to contribute to the diagnosis of the disease process of SSc (113). Lakhter A et al. found that before the onset of T1D, the level of miR-21-5p in serum exosomes would increase progressively, indicating miR-21-5p could be a potential biomarker of T1D (107). Fortunately, miRNAs in exosomes of some AIDs show high specificity and sensitivity for the diagnosis of AIDs disease process (90, 96, 102, 109). However, the sample sizes in these studies were small (N<70), and we should further validate its sensitivity and specificity in a large population before using it as a biomarker of disease activity in AIDs. In addition, in the exosomes of AIDs, the pathogenic mechanism of some miRNAs with high diagnostic value of disease process has not been studied (90, 96, 109). There is no doubt that these pathogenic exosomes provide targets for the treatment of AIDs, but how to identify, isolate and remove these pathogenic “bad” exosomes will become another problem.

#### Treatment and Immunomodulation

On the other hand, due to the unique lipid bimolecular structure, exogenous exosomes can deliver miRNAs from the outside into the organism, and have an intervening effect on the target cells to treat AIDs. In addition to the aforementioned MSC-Exos, other cells-derived exosomes can also deliver miRNAs to treat AIDs. For example, dendritic cell-derived exosomes can deliver miR-146a to promote the formation of Treg cells, ultimately alleviating clinical symptoms in Myasthenia gravis (114).

More importantly, exogenous exosomes can also exert immunomodulatory effects on other cells (115, 116). Relevant studies have shown MSC-Exos could promote the formation of M2 macrophages (117) and regulatory dendritic cells (118) to produce anti-inflammatory effects. Besides, MSC-Exos could inhibit the proliferation and differentiation of T cells (119, 120), B cells (3, 121, 122), and NK cells (123, 124). Interestingly, the biological effects of MSC-Exos on immune cells may be influenced by the microenvironment and ultimately produce different outcomes (125, 126). In addition to MSCs, other cells-derived exosomes can also produce immunomodulatory effects. For example, Exosomes from gene-modified dendritic cells can inhibit the differentiation of Th1 and Th17 cells and promote the formation of Treg cells by delivering TGF-β1 (50). Exosomes derived from B cells (127) and Treg cells (47) can exert anti-inflammatory effects by delivering miR-155 and miR-Let-7d, respectively, which may be helpful for the treatment of AIDs, but further validation is needed. In general, using exosomes to treat AIDs is still in the early stages, because most current studies focused on animal models and experiments *in vitro*.

In conclusion, exosomes are not only involved in the occurrence and development of many AIDs, but also can be used as therapeutic carriers to participate in the treatment of AIDs. On the one hand, studying the mechanism of endogenous exosomes in the occurrence and development of AIDs will help us analyze the reasons for the immune imbalance of AIDs and be beneficial to the diagnosis of the disease process of AIDs. On the other hand, exogenous exosomes have great advantages (low toxicity, stability *in vivo* and high delivery efficiency) in the treatment of AIDs compared to traditional drugs and cell therapy (115, 128), so exploring the therapeutic strategy of exogenous exosomes has great application value for the treatment of AIDs.

### Advantages and Shortcomings

This study has several unique advantages. Firstly, we systematically analyzed research of exosomes in AIDs by bibliometrics for the first time, which can provide comprehensive guidance for scholars who pay attention to related research. Secondly, we used three bibliometric tools simultaneously for the survey, two of which (VOSviewer and CiteSpace) have been widely used in the field of bibliometrics (30), so our data analysis process is very likely to be objective. Finally, bibliometric analysis provides more complete insight into the hotspots and frontiers than traditional reviews.

Of course, this study also has some shortcomings. Firstly, the data of this study are only from the WoSCC database, and other databases are ignored, which may miss some relevant studies. Secondly, we filtered studies published in English, which may mean non-English writing papers were underestimated. What’s more, publications in 2022 were not included due to insufficient data.

## Conclusions

Exosomes have important research value and application prospects in AIDs. The rapidly increasing number of publications shows that the research of exosomes in AIDs is increasingly valued by scholars from the whole world. The leading countries are China and the United States; however, cooperation and communication among various countries and institutions remain to be strengthened. On the one hand, studying the mechanism of endogenous exosomes in the occurrence and development of AIDs will help us to analyze the reasons for the immune imbalance of AIDs and be beneficial to the diagnosis of the disease process of AIDs. On the other hand, compared with traditional drugs and cell therapy, exogenous exosomes have great advantages in the treatment of AIDs, so studying the therapeutic strategy of exogenous exosomes will be of great application value for the precision treatment of AIDs in the future. Notably, in addition to basic research, we should also pay attention to the transformation of research results, that is, the clinical application of exosomes to AIDs patients.

## Data Availability Statement

The original contributions presented in the study are included in the article/supplementary material. Further inquiries can be directed to the corresponding author.

## Author Contributions

FW and JG wrote the manuscript. FW and JG have contributed equally to this work and share first authorship. JK, XW, QN and JL analyzed the data and drew pictures. LZ reviewed and revised the manuscript. All authors read and approved the final manuscript. All authors contributed to the article and approved the submitted version.

## Funding

This work was supported by the National Natural Science Foundation of China [grant number 81771768] and by the applied basic research project of Shanxi Science and Technology Department [grant number 201901D111416].

## Conflict of Interest

The authors declare that the research was conducted in the absence of any commercial or financial relationships that could be construed as a potential conflict of interest.

## Publisher’s Note

All claims expressed in this article are solely those of the authors and do not necessarily represent those of their affiliated organizations, or those of the publisher, the editors and the reviewers. Any product that may be evaluated in this article, or claim that may be made by its manufacturer, is not guaranteed or endorsed by the publisher.
